# Development and validation of a nomogram for predicting survival in patients with malignant myofibroblastic tumor

**DOI:** 10.1002/cam4.5668

**Published:** 2023-03-23

**Authors:** Xiaolu Wang, Baorui Liu, Rutian Li

**Affiliations:** ^1^ The Comprehensive Cancer Centre of Drum Tower Hospital Medical School of Nanjing University & Clinical Cancer Institute of Nanjing University Nanjing China

**Keywords:** malignant myofibroblastic tumor, nomogram, overall survival, prognostic model, SEER

## Abstract

**Background:**

Malignant myofibroblastic tumors are a rare group of soft tissue sarcomas, for which a prognosis prediction model is lacking. Based on the Surveillance, Epidemiology, and End Results (SEER) database and cases from Nanjing Drum Tower Hospital, the current study constructed and validated a nomogram to assess overall survival of patients with malignant myofibroblastic tumors.

**Methods:**

Data of patients with myofibroblastic tumors diagnosed between 2000 and 2018 were extracted from the SEER database. Similarly, data of patients with myofibroblastic tumor in Nanjing Drum Tower Hospital between May 2016 and March 2022 were collected. Then, we conducted univariate and multivariate Cox analyses to identify independent prognostic parameters to develop the nomogram. The model was evaluated by concordance index (C‐index), calibration curve, the area under the curve (AUC), decision curve analysis (DCA), Kaplan–Meier analysis, and subgroup analyses.

**Results:**

Seven variables were selected to construct the nomogram. The results of the C‐index (0.783), calibration curve, the AUCs, and subgroup analyses demonstrated the accurate predictive capacity and excellent discriminative ability of the nomogram. The DCA of the model indicated its better clinical net benefit than that of the traditional system.

**Conclusion:**

Evaluation of the predictive performance of the nomogram revealed the superior sensitivity and specificity of the model and the higher prediction accuracy of the outcomes compared with those of the traditional system. The established nomogram may assist patients in consultation and help physicians in clinical decision‐making.

## Graphical abstract

In the present study, age, grade, tumor size, positive lymph node, N stage, M stage, and chemotherapy were identified as prognostic parameters for survival in patients with malignant myofibroblastic tumors. These factors were included in the construction of the nomogram. Evaluation of its predictive performance implied the superior sensitivity and specificity of the model and the higher accuracy of the outcomes than the AJCC system. The established nomogram may assist patients in consultation and help physicians make appropriate clinical decisions.

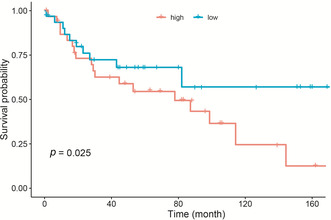

## 1 BACKGROUND

Myofibroblastic tumors represent a rare group of tumors that are observed in both adults and children.^1^, ^2^ These tumors can be classified into subtypes such as myofibroblastic sarcoma (MS), inflammatory myofibroblastic tumor (IMT), myofibromatosis, myofibroblastoma, myopericytoma, and angiomyofibrolastoma. The present study focused mainly on MS and IMT, which are the malignant subtypes. IMT and MS are soft tissue tumors with different malignancy levels. MS is a locally destructive lesion that often recurs and may undergo distant metastases.^3^ IMT is a rare neoplasm with a low incidence of metastasis, a recurrence rate of 2%–25%, and a metastasis rate of approximately 5%.^4^ Due to the rarity of MS and IMT, the recommendations for postoperative adjuvant therapy are lacking, and surgical resection with clear margins remains the preferred treatment.^5^ Abnormal anaplastic lymphoma kinase (ALK) expression has been reported in approximately 50% of patients with IMT. Therefore, ALK inhibitors can be used for IMT patients who cannot be surgically treated.^6^ Evidence for targeted therapy of MS patients is lacking. Moreover, limited clinical data are available on the treatments for both MS and IMT, and little is known about the biological behavior and prognostic characteristics of malignant myofibroblastic tumors, which limits the exploration of the treatment of recurrent or metastatic disease. The present study analyzed the prognostic factors and developed a predictive model for patients with malignant myofibroblastic tumors.

A nomogram is a graphical representation of a statistical prognostic model that involves labeling of variables, which make the assessment of an event probability easier than that with traditional evaluation methods.^7^ This model has been widely used because of the increasing demand for individualized medical treatment for various tumors.^8^, ^9^, ^10^ Therefore, in this study, we developed a model to predict overall survival (OS) of patients with myofibroblastic tumors by using data from the Surveillance, Epidemiology, and End Results (SEER) database and Nanjing Drum Tower Hospital.

## 2 METHODS

### 2.1 Patient selection

**FIGURE 1:**
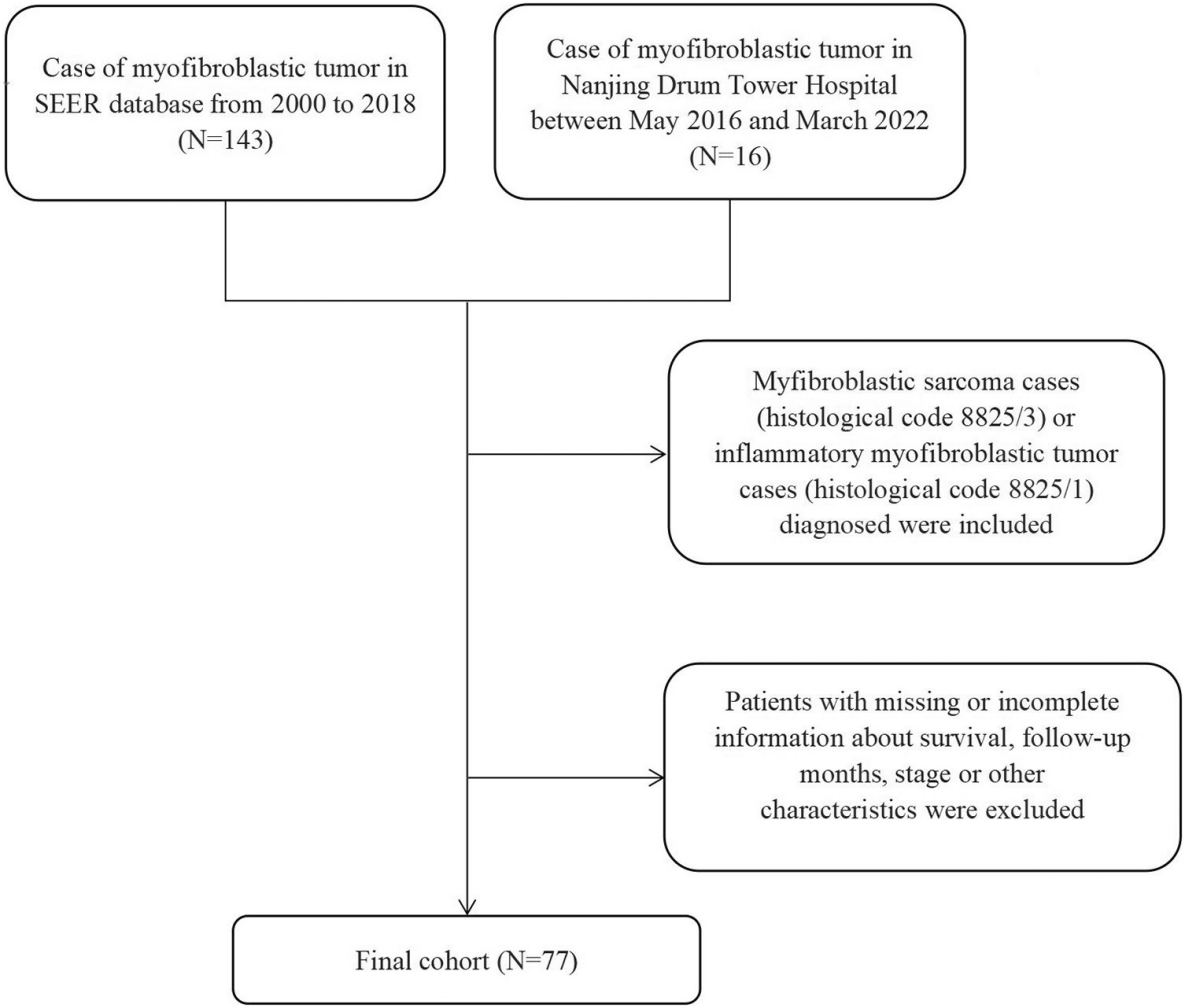
The inclusion and exclusion criteria for selection of the study sample.

Patients with MS (histological code 8825/3) or IMT (histological code 8825/1) diagnosed from 2000 to 2018 in SEER database were enrolled in the study. The SEER database offered demographic and clinicopathological data of the patients. Patients diagnosed with myofibroblastic tumors in Nanjing Drum Tower Hospital between May 2016 and March 2022 were also included. The detailed information of the patients was extracted from the SEER database or medical record system. Our study was conducted in accordance with the guidelines approved by the Ethics Committee of Nanjing Drum Tower Hospital. The parameters in the present study included age, gender, tumor site, tumor grade, tumor size, positive lymph node, 8th American Joint Committee on Cancer (AJCC) tumor (T) stage, 8th AJCC lymph node (N) stage, 8th AJCC metastasis (M) stage, chemotherapy, radiotherapy, surgery, and metastatic status of the bone, brain, liver, and lung. The AJCC TNM system is commonly used to stage soft tissue sarcoma (STS), which is defined by four key components: tumors, lymph nodes, metastases, and grade. Each category (T, N, M and G) is assessed and rated. According to the latest eighth edition of the AJCC staging system, STS can be rated from stages I through IV.^11^ Overall survival (OS) was defined as the period from diagnosis to death or the last follow‐up. In our study, 143 patients who were diagnosed with myofibroblastic tumors were enrolled from the SEER database and 16 patients diagnosed with myofibroblastic tumors collected from medical record system of Nanjing Drum Tower Hospital. After excluding samples with incomplete data, 77 cases were finally included (Figure 1).

### 2.2 Survival analysis

The best cut‐off score was determined by the prognostic index with R, which was utilized to classify the patients with myofibroblastic tumors into high‐ and low‐risk subgroups. Then, survival analysis was performed using the Kaplan–Meier method. Multifactor analysis was conducted, and a multivariate survival model was created to calculate the hazard ratio and confidence interval. “Survival” and “Survminer” R packages in R version 3.6.1 were mainly used in the survival analysis.

### 2.3 Nomogram construction

In total, 16 variables from the SEER database were included: age, gender, tumor site, tumor grade, tumor size, positive lymph node, T stage, N stage, and M stage, chemotherapy, radiotherapy, surgery, and metastatic status of the bone, brain, liver, and lung. The T, N, and M stages were recorded based on the eighth AJCC system. We performed univariate and multivariate Cox proportional hazards regression analyses to identify the parameters associated with prognosis. All the 16 variables were included in the univariate Cox regression analyses. Variables with statistical significance (*p* < 0.05) in the univariate analysis were taken as candidate variables for multivariate Cox regression analyses. By using the “rms” package in R version 3.6.1, we developed a nomogram for prognosis prediction.

### 2.4 Nomogram validation

Due to limited data, internal validation was used in this study according to The Transparent Reporting of a multivariable prediction model for Individual Prognosis Or Diagnosis (TRIPOD) statement.^12^ The concordance index (C‐index) and calibration curve were used to verify the predictive accuracy of the model. For internal validation of the accuracy estimates and to reduce overfit bias, 1000 bootstrap resamples were used. The performance of the model improved with an increase in C‐index (>0.70).^13^ The calibration curve close to the ideal curve exhibited the accurate predictive capabilities of the model.^9^ Additionally, decision curve analysis (DCA) was carried out to visualize the advantages of clinical decision‐making. “rms” and “rmda” packages were mainly used for nomogram validation.

### 2.5 Receiver operating characteristic curve analysis

The time‐dependent receiver operating characteristic (ROC) curves were developed, and the areas under the curves (AUCs) were calculated to investigate the distinction among the 1‐, 3‐, and 5‐year OS nomograms. The AUCs, true‐positive rate (sensitivity or recall), and false‐positive rate (specificity) were determined using a graphical plot. “Survival ROC” R package was mainly used in the ROC curve analysis.

### 2.6 Statistical analysis

All tests were two‐tailed, and a *p* value of <0.05 was considered statistically significant. Statistical analyses were carried out in R statistical software version 3.6.1 (R Foundation for Statistical Computing). All analyses were performed according to the TRIPOD statement (https://www.tripod‐statement.org/).

## 3 RESULTS

### 3.1 Demographics and clinicopathological characteristics

The demographics and clinicopathological features of the patients are displayed in Table 1. Myofibroblastic tumors were observed in the bones and joints (2.6%), trunk or extremities (89.6%), and head and neck (7.8%). The histological types of myofibroblastic tumors were MS (94.8%) and IMT (5.2%). The most common tumor size was ≤5 cm (64.9%), and grade I (36.4%) was the predominant grade. According to the AJCC 8th edition staging system, the percentage of patients with stage I–IV was 18.2%, 22.1%, 18.2%, and 41.6%, respectively. Furthermore, 32.5%, 22.1%, and 29.9% of the patients had undergone radiotherapy, chemotherapy, and surgery, respectively.

**TABLE 1:** Demographics and clinicopathological features of patients with myofibroblastic tumor.

Features	Overall (*n* = 77; %)	SEER cohort (*n* = 70; %)	Hospital cohort (*n* = 7; %)
Age (year)			
≤54	39 (50.6)	35 (50.0)	4 (57.1)
>54	38 (49.4)	35 (50.0)	3 (42.9)
Gender			
Female	38 (49.4)	36 (51.4)	2 (28.6)
Male	39 (50.6)	34 (48.6)	5 (71.4)
Site			
Bones and joints	2 (2.6)	2 (2.9)	0 (0)
Trunk or extremities	69 (89.6)	62 (88.6)	7 (100)
Head and neck	6 (7.8)	6 (8.6)	0 (0)
Grade			
I	28 (36.4)	26 (37.1)	2 (28.6)
II	25 (32.5)	23 (32.9)	2 (28.6)
III	12 (15.6)	9 (12.9)	3 (42.9)
IV	12 (15.6)	12 (17.1)	0 (0)
Tumor size (mm)	59.3 ± 45.7	61.21 ± 47.02	34.71 ± 14.10
T stage			
T1	50 (64.9)	44 (62.9)	6 (85.7)
T2	18 (23.4)	17 (24.3)	1 (14.3)
T3	6 (7.8)	6 (8.6)	0 (0)
T4	3 (3.9)	3 (4.3)	0 (0)
Positive lymph node			
0	51 (66.2)	47 (67.1)	4 (57.1)
1	26 (33.8)	23 (32.9)	3 (42.9)
N stage			
N0	51 (66.2)	47 (67.1)	4 (57.1)
N1	26 (33.8)	23 (32.9)	3 (42.9)
M stage			
M0	66 (85.7)	64 (91.4)	2 (28.6)
M1	11 (14.3)	6 (8.6)	5 (71.4)
Radiotherapy			
No	52 (67.5)	49 (70.0)	3 (42.9)
Yes	25 (32.5)	21 (30.0)	4 (57.1)
Chemotherapy			
No	60 (77.9)	59 (84.3)	1 (14.3)
Yes	17 (22.1)	11 (15.7)	6 (85.7)
Surgery			
No	54 (70.1)	50 (71.4)	4 (57.1)
Yes	23 (29.9)	20 (28.6)	3 (42.9)
Bone metastasis			
No	75 (97.4)	69 (98.6)	6 (85.7)
Yes	2 (2.6)	1 (1.4)	1 (14.3)
Brain metastasis			
No	76 (98.7)	69 (98.6)	7 (100)
Yes	1 (1.3)	1 (1.4)	0 (0)
Liver metastasis			
No	74 (96.1)	69 (98.6)	5 (71.4)
Yes	3 (3.9)	1 (1.4)	2 (28.6)
Lung metastasis			
No	74 (96.1)	69 (98.6)	5 (71.4)
Yes	3 (3.9)	1 (1.4)	2 (28.6)

### 3.2 Prognostic factor identification

**FIGURE 2:**
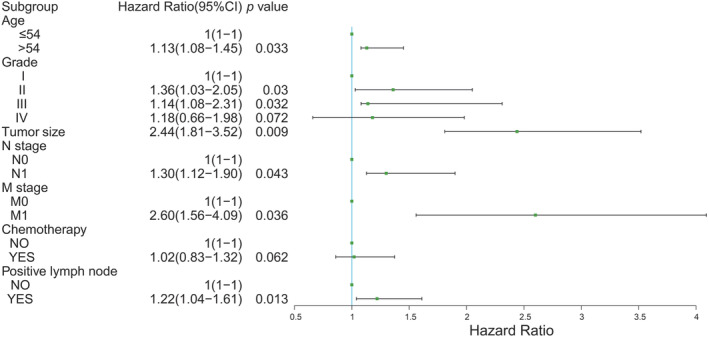
Cox multivariate analysis of the prognostic value of the selected parameters on overall survival of patients with myofibroblastic tumor.

**FIGURE 3:**
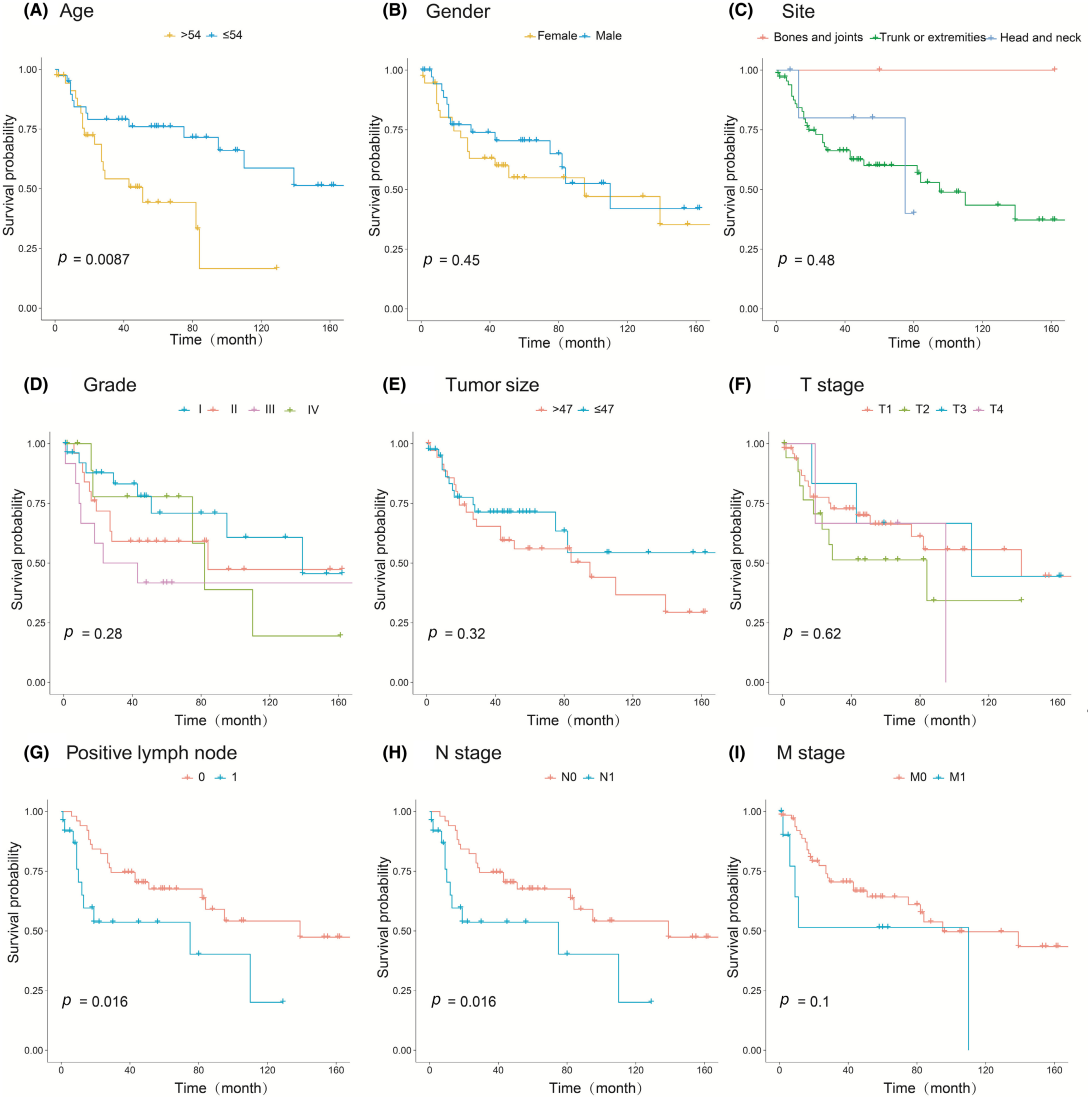
Kaplan–Meier curves for overall survival (OS) of patients with myofibroblastic tumor based on (A) age, (B) gender, (C) site, (D) grade, (E) tumor size, (F) T stage, (G) positive lymph node, (H) N stage, and (I) M stage.

The univariate Cox analysis was performed to explore the impact of demographics and clinicopathological features on survival. Age, grade, tumor size, positive lymph node, N stage, M stage, and chemotherapy were found to be risk factors in patients with myofibroblastic tumor (Table 2). Furthermore, the multivariate Cox analysis results identified age, grade, tumor size, positive lymph node, N stage, M stage, and chemotherapy as independent prognostic factors for survival (Figure 2). Additionally, Kaplan–Meier curve analysis was conducted to demonstrate the prognostic significance of the parameters (Figure 3), suggesting that longer OS was related to younger age (*p* = 0.0087), fewer positive lymph nodes (*p* = 0.016), and lower N stage (*p* = 0.016). On the other hand, gender (*p* = 0.45), tumor site (*p* = 0.48), tumor grade (*p* = 0.28), tumor size (*p* = 0.32), T stage (*p* = 0.62), and M stage (*p* = 0.10) exhibited no significant influence on OS.

**TABLE 2:** Univariate Cox analyses of the factors for the prediction of overall survival of patients with myofibroblastic tumor.

Characteristics	HR	95% CI	*p* value
Age			
≤54	Reference		
>54	1.37	1.17–1.79	0.011
Gender			
Female	Reference		
Male	1.31	0.37–1.54	0.441
Site			
Bones and joints	Reference		
Trunk or extremities	3.75	0.91–4.66	0.879
Head and neck	0.55	0.23–1.37	0.719
Grade			
I	Reference		
II	3.18	1.69–7.12	0.023
III	3.13	2.07–6.18	0.031
IV	2.21	1.36–2.43	0.023
Tumor size	2.76	2.01–5.43	<0.001
T stage			
T1	Reference		
T2	1.36	0.26–3.72	0.013
T3	0.91	0.37–3.22	0.138
T4	1.52	0.69–8.06	0.148
Positive lymph node	1.37	1.09–2.04	0.027
N stage			
N0	Reference		
N1	2.25	1.15–5.21	0.031
M stage			
M0	Reference		
M1	2.89	1.44–4.04	0.034
Radiotherapy			
No	Reference		
Yes	1.18	0.65–2.51	0.090
Chemotherapy			
No	Reference		
Yes	2.44	1.47–6.45	0.015
Surgery			
No	Reference		
Yes	1.28	0.68–2.46	0.451
Bone metastasis			
No	Reference		
Yes	1.45	0.82–6.13	0.564
Brain metastasis			
No	Reference		
Yes	1.66	0.79–3.12	0.643
Liver metastasis			
No	Reference		
Yes	8.35	0.85–13.23	0.056
Lung metastasis			
No	Reference		
Yes	5.27	0.98–8.22	0.051

### 3.3 Nomogram construction

**FIGURE 4:**
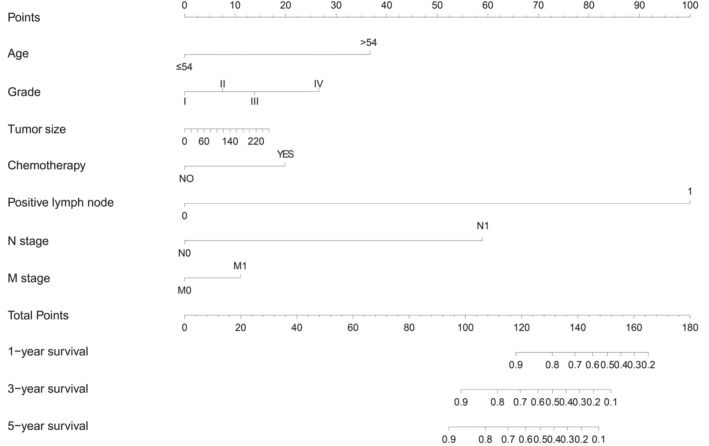
Nomogram for the prediction of 1‐, 3‐, and 5‐year overall survival (OS) in myofibroblastic tumors.

A nomogram containing all independent variables was developed to explore a quantitative method for assessing 1‐, 3‐, and 5‐year OS (Figure 4). The Cox multivariate logistic regression analysis revealed seven significant parameters. The scores of the parameters illustrated in the model were summed up. The number of positive lymph nodes exhibited the greatest impact on the prognosis, followed by N stage and age.

### 3.4 Assessment of predictive accuracy of the nomogram

**FIGURE 5:**
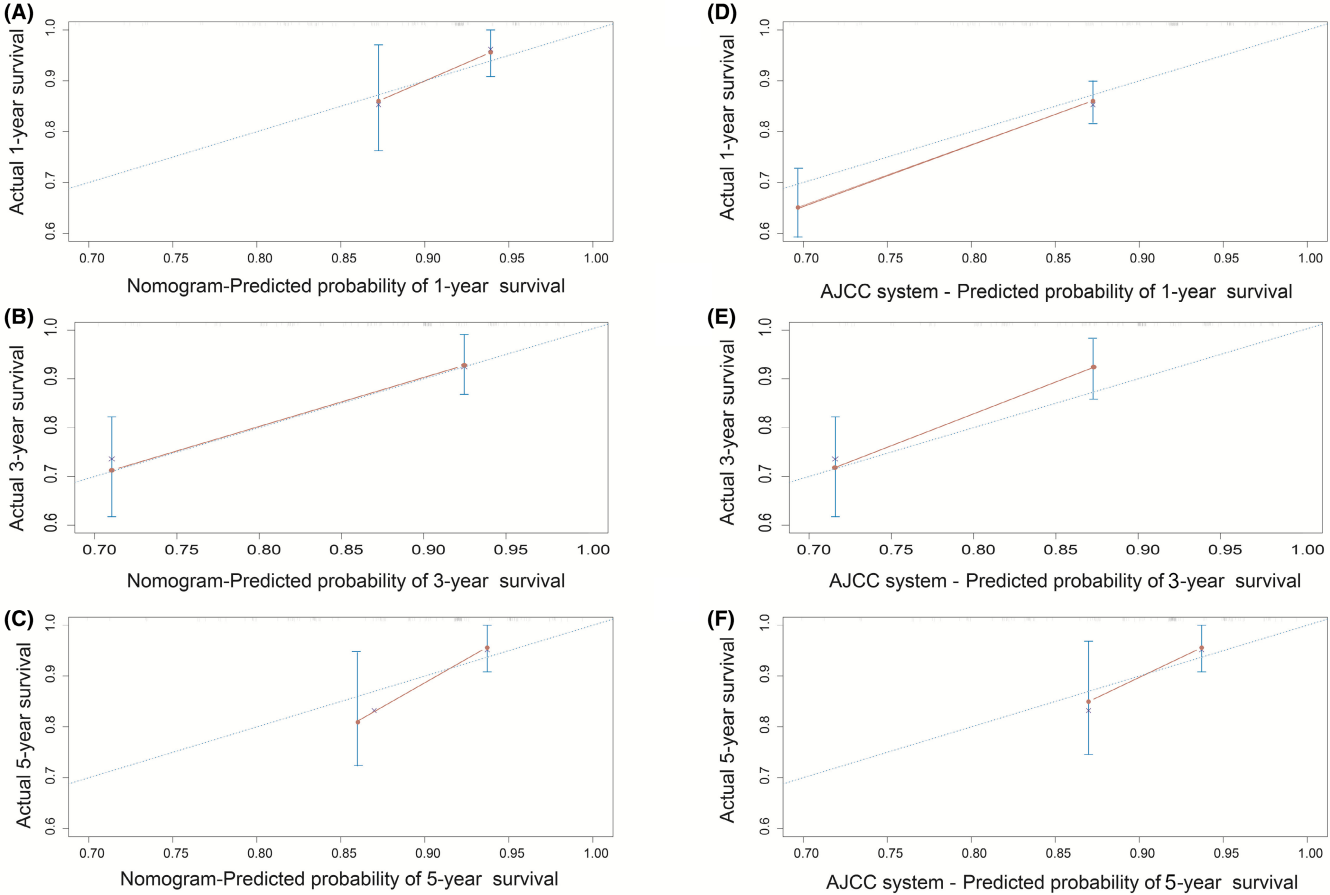
Calibration curves for predicting 1‐, 3‐, and 5‐year overall survival (OS) in myofibroblastic tumors. (A–C) Calibration curve of the nomogram for predicting 1‐, 3‐, and 5‐year OS; (D–F) Calibration curve of the AJCC system for predicting 1‐, 3‐, and 5‐year OS.

**FIGURE 6:**
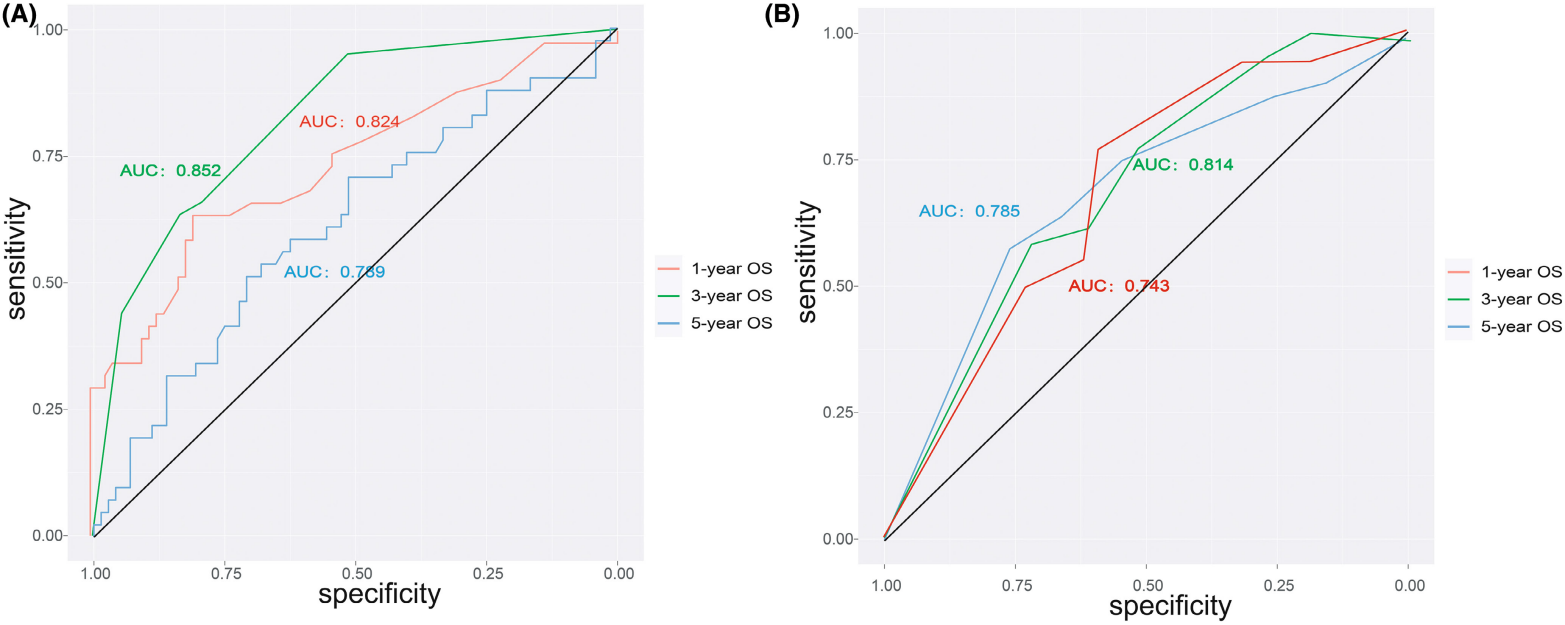
Time‐dependent ROC curves. (A) Time‐dependent ROC curves of the nomogram displayed that the AUCs for predicting 1‐, 3‐, and 5‐year overall survival (OS) were 0.824, 0.852, and 0.789, respectively; (B) Time‐dependent ROC curves of the AJCC system displayed that the AUCs for predicting 1‐, 3‐, and 5‐year OS were 0.743, 0.814, and 0.785, respectively.

We used the C‐index and calibration curves to assess the nomogram and evaluate the predictive accuracy of the model. The C‐indices of the proposed nomogram and the AJCC system were 0.783 and 0.723, respectively, which indicated that the model had higher accuracy than the AJCC system. The degree of agreement observed between the calibration and ideal curves was higher in the proposed nomogram than in the AJCC system (Figure 5). Additionally, the ROC curves indicated that the model has more significant discriminative power than the AJCC system (Figure 6). The AUCs of the nomogram for predicting 1‐, 3‐, and 5‐year OS were 0.824, 0.852, and 0.789, respectively. Nevertheless, the AUCs of the AJCC system for predicting 1‐, 3‐, and 5‐year OS were 0.743, 0.814, and 0.785, respectively. These results showed that the discriminative ability of the nomogram model is superior to that of the AJCC system.

### 3.5 Clinical utility of nomogram

**FIGURE 7:**
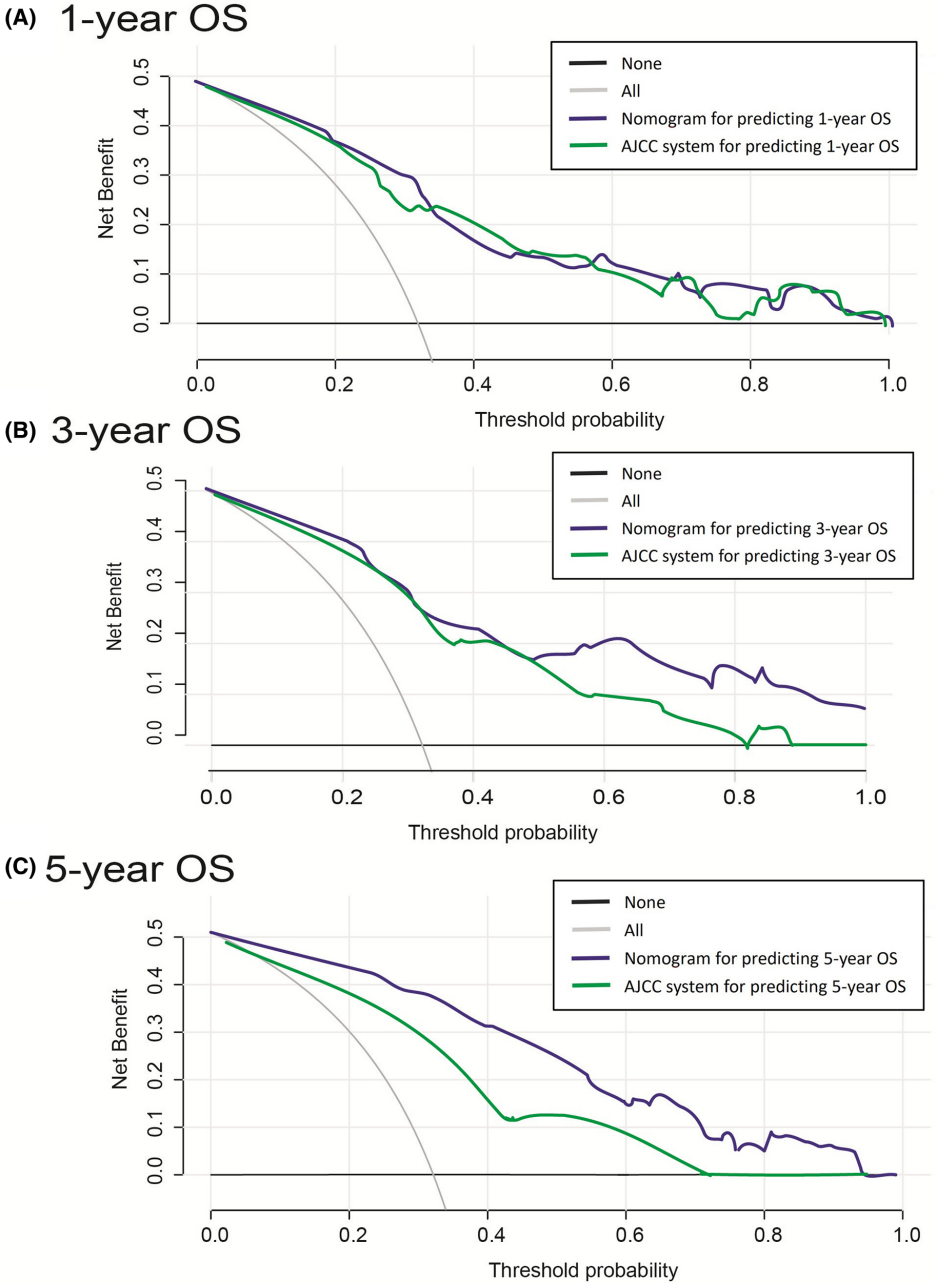
Decision curve analysis (DCA) of the nomogram and 8th AJCC staging system for survival prediction in patients with myofibroblastic tumor. The black line indicates that none of the patients had a high risk of having myofibroblastic tumor, whereas the gray line represents the assumption that all patients had a high risk of having myofibroblastic tumors.

The DCA analysis was performed to assess the clinical significance of the model. The DCA curve illustrated a more comprehensive cut‐off probability range in the newly developed nomogram. In comparison with the AJCC system, the threshold probabilities of the nomogram displayed remarkable net benefits and better performance in predicting 1‐, 3‐, and 5‐year OS of patients with myofibroblastic tumors (Figure 7).

### 3.6 Prognostic score

**FIGURE 8:**
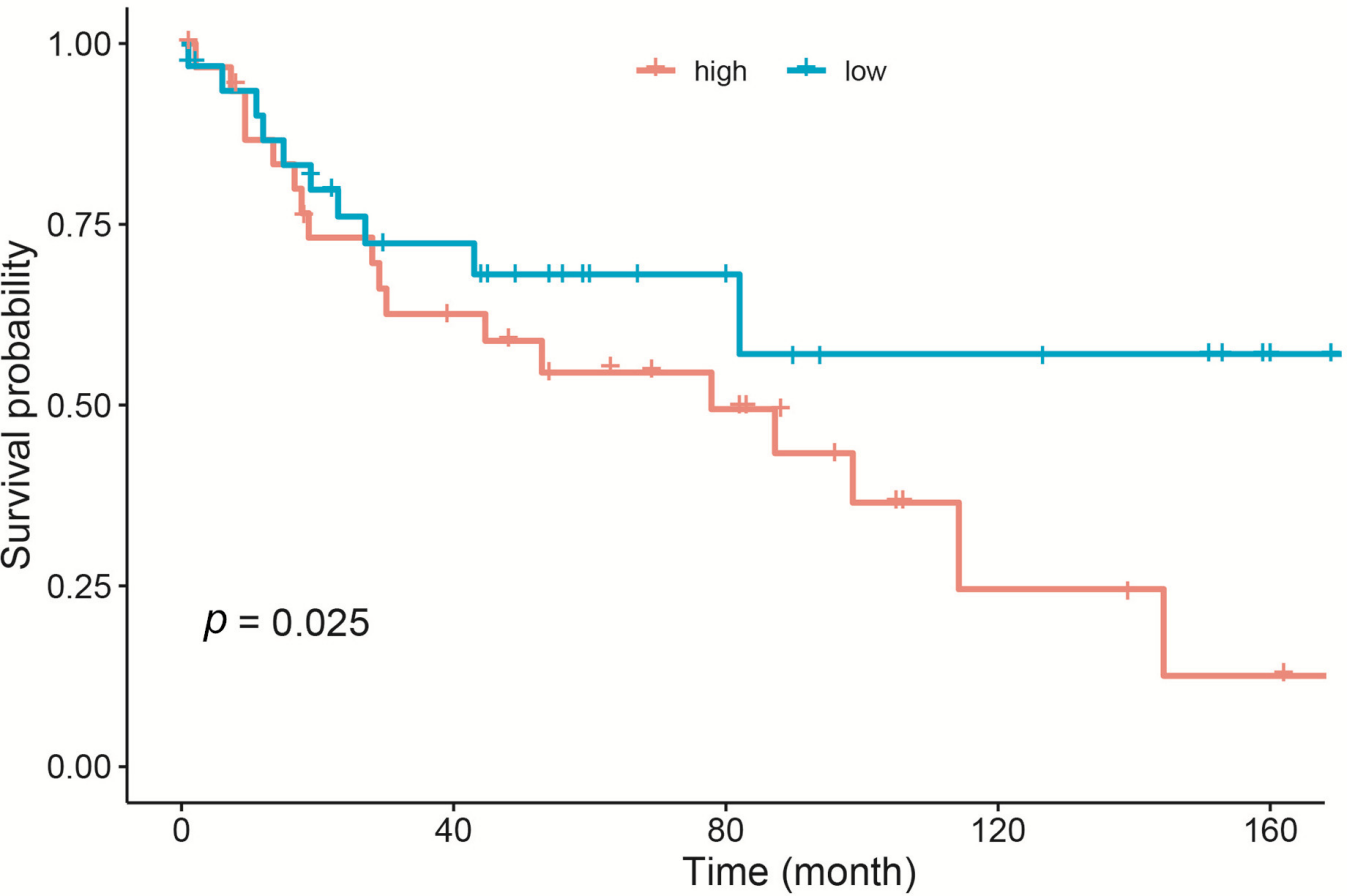
Kaplan–Meier overall survival curves for patients with myofibroblastic tumor in the different risk groups classified by the nomogram.

Finally, risk stratification was performed based on total points calculated using the model. The best cut‐off value was 69 in the new nomogram. The patients with myofibroblastic tumors were classified into two risk groups, namely low‐risk (total points ≤ 69) and high‐risk (total points > 69). The Kaplan–Meier curves exhibited a poor prognosis in patients with the total number of points > 69 (Figure 8). The prognosis differed significantly between the high‐risk group and low‐risk group (log‐rank test; *p* = 0.025).

## 4 DISCUSSION

Myofibroblastic tumors are a rare group of tumors with limited information on prognostic factors. The present study primarily focused on the two major aggressive subtypes MS and IMT. MS is an infiltrative STS mostly involving the head and neck region,^14^, ^15^ which may occur in patients of any age with a slight male predominance.^16^ According to the morphological characteristics, MS can be divided into low‐, intermediate‐, and high‐grades. The World Health Organization (WHO) classified the first two types as low‐grade myofibroblastic sarcoma (LGMS). However, high‐grade myofibroblastic sarcoma (HGMS), also known as myofibrosarcoma multiforme, has not been included in the WHO classification. The recurrence and metastasis rates of HGMS are higher than those of LGMS. IMT is an intermediate STS characterized histopathologically by a heterogeneous group of fibroblasts or myofibroblast spindle cells with infiltration of plasma cells, lymphocytes, and eosinophils.^17^ Due to the different histological appearance of MS, the hypocellular areas of MS may resemble those of IMT. However, IMT exhibits lower cellularity without prominent nuclear atypia and mitosis.^18^ Due to the rarity of the disease, only limited reports on the prognostic factors for malignant myofibroblastic tumors are available. Therefore, an accurate prediction system could be formulated to predict the 1‐, 3‐, and 5‐year OS in patients with malignant myofibroblastic tumor.

In the current study, a personalized nomogram was constructed that integrated conventionally available information such as age, grade, tumor size, positive lymph node, N stage, M stage, and chemotherapy to predict OS in patients with myofibroblastic tumors. Using discrimination, calibration, and clinical utilization analyses as the predictive tool, the nomogram in our study was confirmed to be an accurate and effective model. We used C‐indices, calibration curves, ROC curves, and DCAs to assess our nomogram. The new model provided a better estimation of decision results (net income) than the AJCC system, which is a widely used classification system developed by the American Joint Committee on Cancer for describing the extent of disease progression in cancer patients. In STS, physicians mainly use the AJCC staging system to make preliminary judgments on prognosis. However, due to the wide variety of STS, AJCC staging system is difficult to effectively use to guide all subtypes, especially for rare sarcomas such as malignant myofibroblastic tumors. The proposed nomogram focused on malignant myofibroblastic tumors and showed higher prediction accuracy of the outcomes than the AJCC system. The model also exhibited that patients could be classified into high‐risk and low‐risk groups. The two groups exhibited remarkable differences in survival. The comprehensiveness of the research is a strength of the present study.

Based on standard deviation and nomogram scales, positive lymph nodes emerged as the most crucial prognostic factor, followed by N stage, age, grade, chemotherapy, tumor size, and M stage. Patients with or without positive lymph nodes exhibited a significant difference in survival, indicating the primary influence of lymphatic metastasis on the prognosis of myofibroblastic tumor, similar to most STSs.^19^ Lymphovascular invasion has been reported to be a significant unfavorable pathological factor for STSs in the trunk and extremity^20^; however, lymph invasion was not separately analyzed in the study. In our study, the number of positive lymph nodes was the most significant prognostic parameter, with greater influences on the prognosis than the N stage in the nomogram. The prognosis worsened with positive lymph nodes, probably due to greater risk of metastases in patients with positive lymph nodes; however, the specific mechanism is unknown yet. Future prospective studies are warranted to explore the influence of lymph invasion on patient prognosis.

Patients with STS often experience distant metastasis that are fatal, implying that the prognosis primarily depends on the systemic disease.^21^, ^22^ Patients with metastatic myofibroblastic tumors also seemed to exhibit worse outcomes,^3^, ^23^ the possible reason for which may be that biological aggression plays a major role in prognosis.^24^ However, in our nomogram, the effect of metastasis on prognosis is relatively small. On the one hand, simultaneous or non‐simultaneous resection of the primary tumor and metastases may help improve the prognosis of metastatic myofibroblastic tumors. On the other hand, the small sample size of our study may have affected the accuracy of the conclusion. Thus, more studies are needed to explore the impact of metastasis on the prognosis of myofibroblastic tumors.

In our study, tumor grade was also a crucial prognostic predictor for myofibroblastic tumors. The STS tumor grade was significantly related to the potential for postoperative metastasis and death.^25^ Meng et al. observed that the malignancy of MS increases with advancement of the tumor grade.^3^ These findings are consistent with those of the present study. Additionally, a large tumor size and advanced age were indicators of poor prognosis for myofibroblastic tumors. In addition, a large tumor size may be associated with a high degree of biological malignancy, may require complicated radical surgery, leading to poor quality of life and adverse outcomes. Furthermore, a study on synovial sarcoma revealed that favorable patient outcomes significantly decreased with age regardless of the primary tumor site, tumor size, and treatment.^26^ However, no clear relationship between age and survival has been reported in myofibroblastic tumors.

Our nomogram showed that patients who received chemotherapy exhibited a worse prognosis. Although no standard guidelines exist for the treatment of myofibroblastic tumors, adjuvant therapies such as chemotherapy and radiotherapy can be considered.^27^ Grade II MS exhibits a high recurrence rate and tends to metastasize, and thus, should be treated with surgery and adjuvant therapy,^3^ implying higher tumor malignancy in people receiving chemotherapy. In addition, the sensitivity of myofibroblastic tumors to chemotherapy is controversial,^16^, ^28^ and chemotherapy has a damaging effect on the human body. Future studies are needed to determine whether chemotherapy should be recommended for patients with myofibroblastic tumors, as well as the appropriate regimens and dosages of chemotherapy.

Because the incidence of myofibroblastic tumors is extremely rare, the standardization of its treatments is highly warranted. Curative resection remains the primary treatment modality for myofibroblastic tumors.^29^ According to Meng et al. grade 1 MS should be managed with wide excision and long‐term follow‐up, and grade 2 MS should be managed by excision with a wide margin of normal tissue and adjuvant radiation therapy or systemic chemotherapy.^3^ Casanova et al. reported that IMT patients treated with full surgical resection had excellent outcomes and required no adjunctive therapy.^30^ Limited clinical information is available on the nonsurgical treatment of myofibroblastic tumors. Whether postoperative chemotherapy is effective in malignant myofibroblastic tumors is controversial.^31^ Postoperative radiotherapy has been reported to be potentially effective in MS.^32^ For locally advanced or metastatic myofibroblastic tumors, chemotherapy and radiotherapy can be considered.^29^ Notably, alterations of the ALK gene are the predominant molecular characteristics of IMTs.^33^ The ALK inhibitor crizotinib is, therefore, a recommended targeted agent for the treatment of IMT.^34^ EORTC 90101, a prospective phase II trial evaluating the efficacy of an ALK inhibitor in patients with advanced IMT, showed an impressive ORR of 50.0% for ALK‐positive IMT patients.^33^ Consequently, targeted therapy may affect the prognosis of IMT patients; however, the SEER database did not contain such data. The role of targeted therapy in the prognostic model of myofibroblastic tumor should be further studied. Additionally, medical therapy including the use of cyclo‐oxygenase (COX‐2) inhibitors with or without steroids can be a treatment option for IMT.^35^ Given the rarity of myofibroblastic tumor, no reports on immunotherapy are available. With enhanced understanding of the molecular profile of myofibroblastic tumors in the future, the use of targeted therapy and immunotherapy may increase. However, the optimal treatment strategy for myofibroblastic tumors is yet to be determined.

The present study has certain limitations. First, its retrospective nature and the fact that it was based on large databases might have introduced selection and information biases. Second, detailed clinicopathological information was not provided in the SEER database. Information about chemotherapy and radiotherapy was incomplete, and the reason why some patients did not receive chemotherapy or radiotherapy was unclear. Third, our nomogram can only predict OS for up to 5 years because of the limited follow‐up period. Fourth, the sample size was small due to the rarity of the disease, which may have affected the results. Moreover, because of a small sample size, the current study lacks external validation. More studies are required to confirm the validity of the model. Fifth, the major technological advances made during the period for which data were collected retrospectively may have an impact on the results. However, due to the rarity of myofibroblastic tumors and the slow progress in the development of diagnosis and treatment measures for these tumors, the influence of technological advancements on the results can be considered small. Despite the aforementioned limitations, the population‐based research explored the prognostic parameters of patients with malignant myofibroblastic tumors, thereby confirming the benefits of the nomogram in prognosis prediction.

## 5 CONCLUSION

In the present study, age, grade, tumor size, chemotherapy, positive lymph nodes, N stage, and M stage were identified as prognostic parameters for survival in patients with malignant myofibroblastic tumors. We included these factors in the construction of the nomogram. Evaluation of the predictive performance of the nomogram revealed its superior sensitivity and specificity and higher prediction accuracy of the outcomes compared to the AJCC system. The established nomogram may assist patients in consultation and help physicians make appropriate clinical decisions.

## AUTHOR CONTRIBUTIONS

**Xiaolu Wang:** Conceptualization (equal); formal analysis (equal); investigation (equal); methodology (equal); writing – original draft (equal); writing – review and editing (equal). **Baorui Liu:** Funding acquisition (equal); resources (equal); supervision (equal). **Rutian Li:** Funding acquisition (equal); resources (equal); supervision (equal).

## FUNDING INFORMATION

This work was supported by fundings for Clinical Trials from the Affiliated Drum Tower Hospital, Medical School of Nanjing University.

## CONFLICT OF INTEREST STATEMENT

The authors have no conflicts of interest to declare that are relevant to the content of this article.

## INSTITUTIONAL REVIEW BOARD STATEMENT

Not applicable.

## INFORMED CONSENT STATEMENT

Not applicable.

## ETHICS APPROVAL

This study was approved by the Ethics Committee of Nanjing Drum Tower Hospital (No. 202232601).

## Data Availability

The data used to support the findings of this study are included within the article.
